# Melanocyte lineage dynamics in development, growth and disease

**DOI:** 10.1242/dev.201266

**Published:** 2024-08-02

**Authors:** Alessandro Brombin, E. Elizabeth Patton

**Affiliations:** ^1^ MRC Human Genetics Unit, Institute of Genetics and Cancer, The University of Edinburgh, Edinburgh EH4 2XU, UK; ^2^ Edinburgh Cancer Research, CRUK Scotland Centre, Institute of Genetics and Cancer, The University of Edinburgh, Edinburgh EH4 2XU, UK

**Keywords:** Melanocytes, Melanocyte stem cells, Neural crest, MITF, Pigmentation, Transcriptomics, scRNA-sequencing, Schwann cell precursor, Melanoma, Pigmentary disease, Genomics, Multi-omics

## Abstract

Melanocytes evolved to produce the melanin that gives colour to our hair, eyes and skin. The melanocyte lineage also gives rise to melanoma, the most lethal form of skin cancer. The melanocyte lineage differentiates from neural crest cells during development, and most melanocytes reside in the skin and hair, where they are replenished by melanocyte stem cells. Because the molecular mechanisms necessary for melanocyte specification, migration, proliferation and differentiation are co-opted during melanoma initiation and progression, studying melanocyte development is directly relevant to human disease. Here, through the lens of advances in cellular omic and genomic technologies, we review the latest findings in melanocyte development and differentiation, and how these developmental pathways become dysregulated in disease.

## Teaser

**Summary:** This Review summarises the latest advances in understanding how pigment-producing melanocytes develop from the neural crest, Schwann cell progenitors and melanocyte stem cells, and how these mechanisms are co-opted in melanoma.

## Introduction

Melanin is a complex polymer originating from the amino acid tyrosine that produces a brown-black pigment, which absorbs and reflects different wavelengths of light, giving colour to skin, eyes and hair in humans and animals (Mort et al., 2015). Melanin is produced in lysosome-like organelles called melanosomes in a specialised cell type, called melanocytes, and provides protection from UV radiation (Benito-Martinez et al., 2021). Mammalian melanosomes are distributed to the surrounding skin keratinocytes, resulting in even skin tone (Le et al., 2021). Similarly, in the hair follicle, melanocytes transfer pigment to the growing hair shaft to provide colour. Other melanocytes, with less well-understood functions, are also found, e.g. in the heart and inner ear (Brito and Kos, 2008; Gudjohnsen et al., 2015; Tachibana, 1999; Yajima and Larue, 2008).

**Fig. 1.**
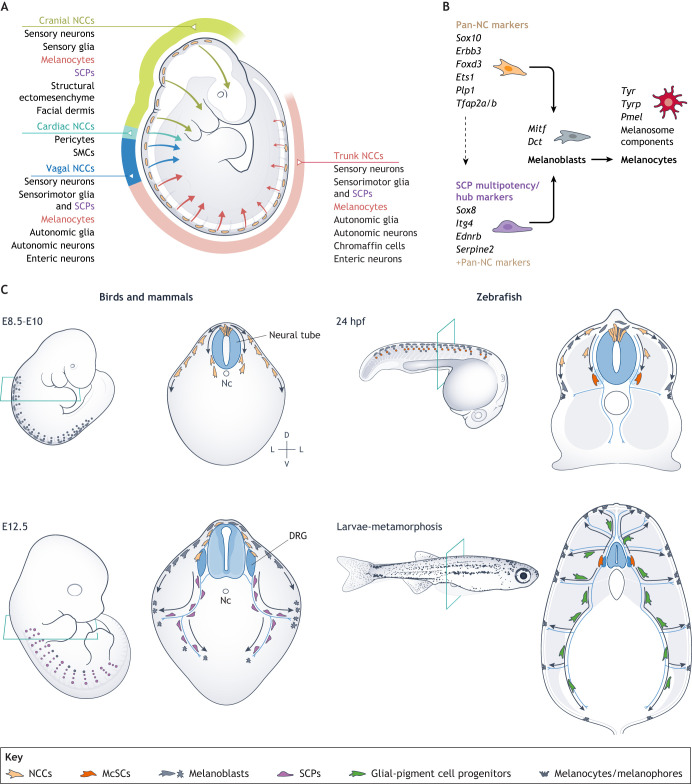
**Melanocyte origins in development.** (A) Neural crest cells (NCCs) are stem-like cells that generate a multitude of cell types, including derivatives in the cranial, vagal and trunk regions of the embryo. Cranial neural crest derivatives include facial ectomesenchyme (facial bones and cartilages), sensory nerves and facial dermis. Cardiac neural crest derivatives include pericytes and smooth muscle cells (SMCs). Vagal neural crest and trunk neural crest derivatives are similar and include different types of neurons (motor, sensory and enteric) and glia. (B) Upon induction, NCCs express a set of well-defined markers. The same markers are expressed, in mouse, later in development by the axon-associated SCPs together with the multipotency ‘hub’ markers (*Sox8*, *Itg4*, *Ednrb* and *Serpine2*) that keep the SCPs in a multipotent state primed for differentiation. The key event in melanocyte differentiation is the expression of the melanocyte-inducing transcription factor (*Mitf*), which triggers the expression of the Mitf-target genes involved in the synthesis of melanin and/or the formation of the melanosomes. (C) In mouse, bird and zebrafish experimental models, skin melanoblasts and melanocytes originate early in development from migrating NCCs (top) and later from progenitors associated with peripheral nerves (bottom). D, dorsal; DRG, dorsal root ganglion; L, lateral; McSC, melanocyte stem cell; Nc, notochord; V, ventral.

**Fig. 2.**
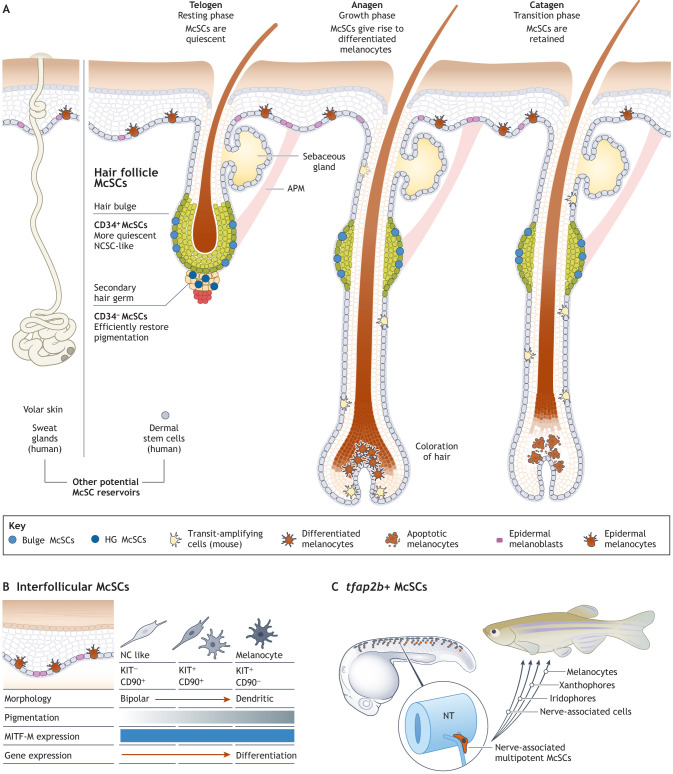
**Melanocyte stem cell populations.** (A) Melanocyte stem cells (McSCs) are found in a variety of locations in the mammalian skin, including in the hair follicle in both mouse and human. Each hair follicle is a tube-like structure that supports a single hair and goes through cycles of growth. Each cycle consists of three phases: telogen (resting phase), anagen (a proliferative phase) and catagen (transition stage). McSCs can be detected in the hair bulge area and in the hair germ (HG) during the telogen phase. McSCs in HG go through a transit amplifying-like stage in early anagen, and contribute to both differentiated melanocytes and renewal of McSCs in both compartments with each hair cycle (Sun et al., 2023). Hair follicle McSCs can also contribute to epidermal melanocytes (Chou et al., 2013). Fully differentiated melanocytes in the hair bulb contribute to hair coloration during late anagen before dying during catagen. Moreover, McSCs can be found in the sweat glands in volar skin (palms and soles), and, potentially, melanocytes could originate from dermal stem cells in the human dermis. (B) Recently, three cell states of melanocytic cells, including pigmented and dendritic cells, and two populations of undifferentiated cells (represented as purple cells in the schematic) have been described in the interfollicular regions of human skin. (C) In zebrafish, McSCs are associated with the site of the dorsal root ganglia (DRG) and transiently express *tfap2b* during establishment in the niche. These *tfap2b*^+^ cells reside in the McSC niche and lineage tracing studies demonstrate that they can generate all three pigment cell types and nerve-associated cells in the adult animal. APM, arrector pili muscle; MITF-M, microphtalmia-associated transcription factor (melanocyte-inducing transcription factor)-M; NT, neural tube; NCSC, neural crest stem cells.

Melanocytes differentiate early in development from progenitors called ‘melanoblasts’, which are derived from neural crest cells (NCCs), a transient and migratory cell population with stem cell properties. NCCs originate at the border of the neuroectoderm during the closure of the neural tube and generate a wide range of different cell types (Erickson et al., 2023) (Fig. 1). After birth, however, melanocytes are replenished in mammals by melanocyte stem cell (McSC) populations residing in the hair follicle, the dermis and the sweat glands on the palms and soles (Nishimura et al., 2002; Okamoto et al., 2014; Zabierowski et al., 2011) (Fig. 2). Melanocytes can also be replenished in chick and mouse upon nerve injury by Schwan cell precursors (SCPs), which are multipotent, peripheral nerve-associated progenitors that are also derived from NCCs (Adameyko et al., 2009; Nitzan et al., 2013).

Understanding the developmental biology of melanocytes is important for human health because the developmental pathways necessary for melanocyte specification from either NCCs or McSCs are often co-opted and repurposed in melanoma initiation, progression and for the acquisition of drug resistance (Belote et al., 2021; Diener and Sommer, 2021; Gopalan et al., 2022 preprint; Johansson et al., 2020; Kaufman et al., 2016; Konieczkowski et al., 2014; Marie et al., 2020; Rambow et al., 2018; Shakhova et al., 2012; Travnickova et al., 2019; Varum et al., 2019; White et al., 2011). Thus, the study of the co-opted developmental mechanisms provides a rich source of novel drug leads and targets for the next generation of melanoma therapies (Patton et al., 2021). Moreover, information on how McSCs are maintained and reactivated could help highlight previously unreported developmental mechanisms contributing to pigmentary syndromes and with therapeutic potential in regenerative medicine.

Here, we focus on studies published since our previous Review (Mort et al., 2015), and review how recent advances in sequencing technologies, in particular multi-omic strategies including single-cell transcriptomics, genomics and epigenomics, have provided new insight into the biology of the melanocyte lineage in development and disease.

## Melanocyte origins in embryogenesis

### Neural crest cells as the origin of melanocytes

During neurulation, NCCs undergo an epithelial-to-mesenchymal transition within the dorsal neural tube to delaminate from the epithelium with anterior-to-posterior developmental asynchrony (Artinger and Monsoro-Burq, 2021; Kotov et al., 2024; Rothstein et al., 2018). NCCs then migrate to distinct locations within the embryo, where they differentiate into different cell types, including neural, melanocyte, cartilage and bone cells (Fig. 1A) (Tang and Bronner, 2020). Cranial NCCs are the first to be specified and migrate from the region spanning the hindbrain and the anterior neural tube, producing ectomesenchymal derivatives in the head and developing face, such as bones and cartilages, dermis and tooth pulp. Uniquely, cranial NCCs give rise to multiple, spatially defined overlapping oligopotent clonal patches called ‘clonal envelopes’ (Kaucka et al., 2016). In contrast, vagal, cardiac and trunk NCCs migrate in streams and differentiate into sensory, autonomic and enteric neurons, glia, SCPs, melanocytes and chromaffin cells (Rothstein et al., 2018) (Fig. 1A).

Whether all NCCs are multipotent and when they become restricted for specific fates *in vivo* are key questions in the NCC field. Given the differences in methodological approaches, species, developmental stages and axial NCC location, these questions have remained difficult to answer (Erickson et al., 2023). However, evidence from single-cell RNA sequencing (scRNA-seq) experiments from zebrafish, *Xenopus*, chick and mouse suggest that, early in development, NCCs may be in a state uniquely primed for specific fates while retaining a high degree of multipotency (Baggiolini et al., 2015; Bronner, 2015; Ji et al., 2021 preprint; Kaucka et al., 2016; Kotov et al., 2024; Lencer et al., 2021; Lukoseviciute et al., 2018; Perera and Kerosuo, 2021; Soldatov et al., 2019; Tatarakis et al., 2021; Williams et al., 2019).

How NCCs subsequently commit to a specific lineage has been explored in a landmark scRNA-seq study of trunk murine NCCs, whereby the developmental asynchrony in the anteroposterior axis has been leveraged to capture downstream NCC trajectories and fates (Soldatov et al., 2019). Combined with spatial transcriptomics, this approach has identified distinct states that are spatially segregated in the embryo. During NCC differentiation, binary fate decisions are preceded by a ‘biasing stage’, with broad shifts in gene expression between competing fate programmes. These progressive binary choices between alternative fates drive NCCs towards autonomic, sensory or mesenchymal fates.

Surprisingly, a specific developmental branch for melanocyte development in either the trunk or cranial neural crest (NC) has not been found (Kastriti et al., 2022; Soldatov et al., 2019). In support of a pan-NC origin of the developing melanocyte lineage, melanoblasts emerge sporadically from different branches of the NC specification ‘tree’ through the expression of Mitf, the master melanocyte-inducing transcription factor (Goding and Arnheiter, 2019) (Fig. 1B). Among the NCCs, *Mitf* expression is inhibited by different transcription factors, such as FoxD3, preventing sufficient levels of Mitf to accumulate to coordinate the downstream melanocyte lineage programme (Kos et al., 2001; Lukoseviciute et al., 2018; Thomas and Erickson, 2009). In the zebrafish NC, Foxd3 first acts as a transcriptional repressor but later switches to become an activator to promote NC fates (Lukoseviciute et al., 2018). Thus, rather than emerging from a binary fate decision, melanocyte precursor cells emerge from different NC streams as soon as *Mitf* transcription is no longer inhibited. This process is termed ‘fate convergence’, meaning that distinct progenitor pools of different lineages ultimately contribute to the same cell type. In one example of this pan-NC origin for melanocytes, downregulation of *Neurog2* in the delamination stage from cells of the sensory lineage led to an increase in Mitf and the acquisition of a melanocytic fate (Soldatov et al., 2019).

Zebrafish have three pigment cell types, all of which derive from the NC: black/brown melanocytes, yellow xanthophores and iridescent iridophores. Although some embryonic yellow xanthophores directly emerge from the NC (Mahalwar et al., 2014; McMenamin et al., 2014), others emerge via tripotent and bipotent intermediate progenitors all expressing *mitf* (*mitfa* in zebrafish) (Bagnara et al., 1979; Brombin et al., 2022; Brunsdon et al., 2022; Kelsh et al., 2021; Petratou et al., 2021, 2018; Subkhankulova et al., 2023). In this context, Foxd3 is involved in repressing *mitfa* in melanocyte-iridophore bipotent progenitors and promoting iridophore production (Curran et al., 2010, 2009). Transcriptional diversity among pre-migratory trunk NC populations has been identified through scRNA-seq, with genes associated with differentiated derivatives, specifically in the xanthophore lineage, already expressed (Lencer et al., 2021). However, the consequence of the transcriptional diversity seems to differ from that of cranial NC, as scRNA-seq on the first pharyngeal arch provided evidence that melanocytes directly emerge from the NCCs, without evidence of heterogeneity and before specification of the glial lineage (Tatarakis et al., 2021).

Recently, it has been shown that the tyrosine kinase-coding gene *ltk* acts in a bimodal fashion during iridophore differentiation. Specifically, progenitors expressing markers for all pigment cells and neural derivatives have been found in both premigratory and migrating NCCs by combining NanoString and RNAscope technologies. These data support a model in which migrating NCCs remain multipotent and do not restrict their fate through binary choices (Kelsh et al., 2021; Subkhankulova et al., 2023). Data from scRNA-seq data from a 24 h zebrafish embryo (at a time when NCs are migrating, with some already specified) support the concept that a subpopulation of embryonic NCs expresses all three pigment cell genes and glial genes (termed MIX+), proposed as multi-potent pigment cells that emerged directly from the NC (rather than via an ERB-dependent McSC population; see below) (Brombin et al., 2022). Thus, at least in the context of trunk NC, zebrafish NCCs may be multipotent cells.

The specification of melanocytes from NCCs requires extra levels of regulation that MITF activity alone cannot provide. MITF acts as a dose-dependent ‘rheostat’ such that lower levels of Mitf activity promote proliferation, whereas higher levels of Mitf activity promote differentiation, both in development and melanoma progression, owing to the activity of transactivating factors and changes in DNA affinity promoted by histone variants or acetylation of the protein itself (Louphrasitthiphol et al., 2023, 2020; Miyadai et al., 2023; Raja et al., 2020; Taylor et al., 2011; Zeng et al., 2015). In zebrafish, the Mitf-related protein Tfec specifies early pluripotent pigment cell progenitors from migrating NC cells and later promotes iridophore fate (Petratou et al., 2021). Among genes with dual roles in both NC and melanocyte specification are the Tfap2 genes (transcription factor alpha protein 2). Early in development, Tfap2 genes are expressed in the neural plate, where they contribute to the specification of pre-migratory NC (Williams et al., 2019). Interestingly, Tfap2 genes are then re-expressed during specification and differentiation of melanocytes (Simoes-Costa and Bronner, 2015). In zebrafish, Tfap2 proteins regulate *sox10* expression (Van Otterloo et al., 2012), and melanocyte differentiation gene expression is Tfap2a dependent in both fish and mouse (Seberg et al., 2017). Applying scRNA-seq and ATAC-seq, Tfap2 proteins have been shown to act as pioneer transcription factors and to open the chromatin for *Mitf(a)* binding sites (Kenny et al., 2022). Further studies in zebrafish using single-embryo 3' transcriptional profiling coupled with RNA-seq have identified a core gene regulatory network of genes for NC, regulated by Tfap2c (Dooley et al., 2019). Thus, Tfap2 proteins play a crucial role in the transition from NC to melanocyte fate.

### SCPs as a source of melanocytes

Although ample evidence supports the current theory that melanocytes develop directly from NCCs in the early developing vertebrate embryo, melanocyte production at later developmental stages is not well understood. SCPs could serve as a secondary source of melanocytes because when SCPs detach from the distal end of nerves, they can differentiate into melanocytes (Adameyko et al., 2009, 2012). This source of melanocyte progenitors is activated upon wounding and repair. SCPs also contribute to melanocytes in the ventral region of the developing chick embryo (Nitzan et al., 2013). Nevertheless, their full contribution in mammals has remained somewhat elusive due to differences in lineage tracing experimental design (see Vandamme and Berx, 2019).

Murine SCPs have the potential to generate different cell types, including melanocytes (Debbache et al., 2018). Moreover, during development, scRNA-seq of NCCs and SCPs has revealed that they pass through a communal multipotent transcriptional landscape, called a ‘hub state’, in which a primed state, characterised by expression of *Sox10*, *Foxd3*, *Tfap2a* and *Tfap2b*, together with *Sox9* and *Ets1*, enables quick production of NC derivatives, including the melanocytes. In contrast, *Serpine2*, *Itga4*, *Ednrb*, *Dlx1*, *Dlx2* and *Sox8* maintain a quiescence state (Kastriti et al., 2022) (Fig. 1B).

Experimental evidence of SCP capacity to produce melanocytes in mammals in recent lineage-tracing studies supports the observation that SCPs are the cellular origin of extracutaneous melanocytes in the inner ear, heart and supraorbital locations (Kaucka et al., 2021). Furthermore, SCPs acquire a melanocytic fate in the limbs after β-catenin activation, which induces the expression of microphthalmia transcription factor isoform M (Mitf-M) and represses *Foxd3* (Colombo et al., 2022). This mechanism may also be conserved in humans because scRNA-seq analysis of Schwann cells generated from human pluripotent stem cells has shown that Schwann cells cluster with cells of melanocyte identity, suggesting they share a common progenitor (Majd et al., 2023). Together, the multi-omic data combined with the experimental evidence indicate that SCPs have the potential to generate melanocytes (Fig. 1B,C).

It seems likely that the SCP capacity to produce melanocytes is not limited to amniotes. As discussed above, after NCC migration and in postembryonic stages, the axons of the peripheral nerves in zebrafish are lined by precursors that contribute to the generation of the stripe melanocytes once the fish undergo metamorphosis (Brombin et al., 2022; Budi et al., 2011; Dooley et al., 2013; Singh et al., 2016). Labelling these cells using the NCC-specific *sox10:cre^ERt2^* transgene has shown that, similar to mammalian SCPs, these cells can give rise to all three pigment cell types and label nerve cells (Singh et al., 2016). Transcriptional profiling of *sox10:cre^+^* cells at different developmental stages has demonstrated that axon-associated pigment progenitors are similar to SCPs (Saunders et al., 2019) (Fig. 1C). The creation of new mammalian datasets from mouse development and human-induced pluripotent stem cells (Gopalan et al., 2022 preprint; Kastriti et al., 2022; Majd et al., 2023) now offer the potential to perform comparative transcriptomic analyses with birds and fish, and determine whether the SCP contribution to the melanocyte complement is conserved across evolution.

### Potential other sources of melanocytes?

In the previous sections, we analysed how melanocytes are specified early in development, but the story might not be complete. In recent years, the analysis of birthmarks of people living with congenital melanocytic disorders suggests that melanocytes might originate from unexpected origins. This novel hypothesis is discussed in Box 1.

**Box 1. Additional embryonic origins of melanocytes**

Genetically mosaic human congenital pigmentation disorders affecting the skin, including pigmented lesions and vitiligo, can form segmental patterns that are consistent with the classical understanding of a neural crest origin of melanocytes (Kinsler and Larue, 2018; van Geel et al., 2013). However, many mosaic congenital pigmentation disorders are not consistent with this segmental pattern, suggesting additional, yet unknown, sources of melanocytes. Kinsler and Larue (2018) performed a systematic review of over 6400 photographs of 1229 patients with congenital pigmentation disorders that affect the skin and identified three recurrent patterns: segmental pattern (‘quadrilateral shaped’); non-segmental pattern (‘round shaped’); and broad Blaschko-linear patterns (considered to be non-cell autonomous). These patterns were not due to specific genetic mutations, because they were found across genetically distinct mosaic diseases. Kinsler and Larue proposed that the melanocytes that expand to form pigmentary lesions in a ‘round shaped’ pattern might originate developmentally from uncharacterised progenitors. This melanoblast population colonises the head, trunk and proximal limbs bilaterally and symmetrically, both dorsally and ventrally. In addition, ‘round-shaped’ pattern pigmentary disorders have predominantly dermal histological pathology and do not affect key epidermal structures (e.g. lips and nipples). Thus, there may be some melanocytes that are not derived directly from NCCs and instead migrate to the dermis via an undescribed developmental pathway, or possibly even via SCPs, but further research is needed to confirm this hypothesis.

## Melanocyte stem cells

McSC populations are established in the embryo, with the potential to replenish melanocytes during adult life, as well as in diseases such as vitiligo.

### Hair follicle McSCs

Our understanding of melanocyte biology has been transformed with the discovery of McSCs in the bulge of the outer root sheath of the hair follicle (Fig. 2A). These McSCs can be detected in the hair germ (sub bulge region) during the telogen phase (resting phase) (Nishimura, 2011; Nishimura et al., 2005, 2002, 2010) (Fig. 2A). These slow-cycling McSCs reside with the hair follicle stem cell and can be labelled using the *Dct:LacZ* transgene. During hair growth (anagen), McSCs generate melanocyte progenitors that migrate down the hair shaft to the bulb area, where they differentiate and pigment the growing hair shaft in the inner core of the hair matrix (Fig. 2A). Exhaustion of McSCs leads to hair greying during ageing and can be accelerated by DNA damage, such as irradiation, which leads to premature differentiation in the niche (Inomata et al., 2009; Nishimura et al., 2005).

### Melanocyte stem cell exhaustion

In recent years, the ability to isolate viable McSCs using FACS, combined with RNA-seq, has been a powerful strategy for understanding McSC renewal and differentiation, revealing new mechanisms of hair greying. For example, McSCs from the hair follicle in mice haploinsufficient for MITF activity are sensitised to premature hair greying (Harris et al., 2018). RNA-seq of McSCs from these mice showed that although pigmentation gene expression is reduced in MITF heterozygous animals, there is also an activation of a type I viral innate immunity signature of interferon-stimulated genes (Harris et al., 2018). This is especially interesting given that previous work had shown that after sunburn in neonates, melanocytes are activated by IFN-γ from macrophages, which activates melanocytes to express an IFN gene signature, including for immuno-evasion (Zaidi et al., 2011). Indeed, Harris and colleagues have shown by ChIP-seq that MITF repressed systemic innate immune gene expression. Innate immune signalling induced by systemic exposure to the viral mimic poly (I:C) is sufficient to promote hair greying in genetically sensitised animals, reducing McSCs and increasing differentiated melanocytes in the bulb. Although uncommon, there are examples of people developing grey hair after viral infection or interferon treatment, suggesting transient or age-related reductions in MITF suppression of an innate anti-viral immune response, the activation of which is detrimental to melanocyte and McSC maintenance, might be responsible for hair greying in some people (Harris et al., 2018). In addition, in mice, sympathetic nerves that innervate the hair follicle during stress release noradrenaline to promote hyperproliferation and subsequent depletion of McSCs, explaining how high levels of stress might lead to premature hair greying (Zhang et al., 2020).

To gain insight into temporal mechanisms that define the differentiation of mouse McSCs, transgenic markers combined with carefully timed sampling during the hair follicle cycle have been used to capture quiescent McSCs, activated McSCs and proliferative progeny for scRNA-seq analyses. This strategy has revealed that activation of McSCs is dependent on Wnt signalling, whereas BMP signalling (in activated McSCs) promotes their differentiation to fully mature melanocytes (Infarinato et al., 2020). Generating conditional lineage-specific genetic ablation of *Bmpr1a* has shown that, although McSCs remain intact, mice undergo premature hair greying because BMP signalling is required for the expression of melanosome components during the transition from activated McSC to melanocyte maturation.

### Two populations of melanocyte stem cells in the hair follicle

Whether bulge and hair germ McSC populations are functionally distinct has been unclear. These two McSC populations can be physically distinguished by CD34 antibodies: bulge McSCs express CD34, whereas hair germ McSCs are negative for CD34. FACS analysis in an inducible transgenic mouse engineered to express GFP under the control of the *Dct* promoter (*Dct:H2BGFP* containing the transgenes *Dct:tTA* and *TRE-H2BGFP*) (Joshi et al., 2018) captured CD34^+^ and CD34^−^ McSCs, representing the bulge and hair germ cells, respectively (Joshi et al., 2019). RNA-seq analyses have revealed the transcriptomic differences between these populations: CD34^−^ McSCs express higher levels of genes associated with melanocyte differentiation, whereas CD34^+^ are more similar to neural crest stem cells (NCSCs), including the expression of glial markers. In addition, the two McSC populations are functionally distinct: CD34^−^ McSCs regenerate pigmentation more efficiently, whereas CD34^+^ McSCs selectively myelinated neurons, suggesting that bulge CD34^+^ cells have multipotent progenitor cell properties associated with the NCSCs (Joshi et al., 2019). Notably, both populations are capable of populating both niches, suggesting plasticity (Fig. 2A).

In a recent tour-de-force, a combination of scRNA-seq analysis and live imaging of permanently labelled McSCs over 2 years in the mouse hair germ has revealed that, contrary to other stem cell systems, hair follicle McSCs move during the hair cycle (Sun et al., 2023). These upward and downward movements are associated with cycles of McSC differentiation (including expression of pigmentation genes) and subsequent de-differentiation into hair germ McSCs, contrasting with other tissue stem cell populations in which the stem cells are confined to their niche. Not all cells that move to the hair bulge return to the hair germ layer at every cycle. Indeed, over time, more McSCs are found in the CD34-negative compartment in the hair bulb (and in general in more distant areas of the hair shaft) in aged mice compared with young mice. These observations could explain the mechanisms underlying age-related hair follicle McSC depletion and hair greying in mammals (Sun et al., 2023) (Fig. 2A).

Taken together, mouse hair follicle McSCs appear to be a highly dynamic population, responding to both internal and external stresses. These studies likely reflect human biology; in *ex vivo* cultured human hair follicles, McSCs also respond to stress by ectopic pigmentation and differentiation in the niche, indicating that the human and murine McSCs undergo similar phenotypic responses to stress (Rachmin et al., 2021). Generally, similar McSC populations have been described in both murine and human hair follicles (Cui et al., 1991; Nishimura, 2011; Nishimura et al., 2002; Schneider et al., 2014). In the future, scRNA-seq and other multi-omic datasets from the human hair follicle (including those from Takahashi et al., 2020; Wu et al., 2022) will allow cross-species comparisons and a refined molecular description of the human follicular melanocytic population.

### Skin McSC populations

Although some interfollicular melanocytes are replenished by follicular McSCs (Nishimura, 2011), little is known about the cells responsible for melanocyte replenishment in most of the interfollicular space. In mouse skin, interfollicular melanocytes are largely absent, but interfollicular McSCs have been described in the mouse tail, where the skin-covered protrusion has a pigmented interfollicular epidermis in addition to pigmented hairs (Glover et al., 2015). Interfollicular melanocytes may also originate from dermal stem cells in glabrous skin (Li et al., 2010) and in sweat glands (Okamoto et al., 2014); both can produce melanocytes *in vitro* and could represent melanocyte reservoirs *in vivo*.

In a recent study of human skin, three MITF-M-expressing and phenotypically distinct populations of pigmented melanocytic cells have been identified based on co-expression of the stem cell maker CD90, and the melanocyte-specific marker KIT. Residing in the basal cell layer of the epidermis, these populations include pigmented, poly-dendritic (‘star-shaped’) differentiated melanocytes, mixed bipolar and dendritic cells with less pigmentation, and bipolar cells with almost no dendrites or pigmentation (Michalak-Micka et al., 2022) (Fig. 2B). The authors based their experimental work on earlier studies that identified three distinct populations of melanocytes in the human scalp skin that could be identified by morphology and cell markers (Klar et al., 2017; Tobin and Bystryn, 1996), and also found these cells in the foreskin, eyelid, palm, ear and abdomen skin regions. RNA-seq has revealed that the ‘star-shaped’ differentiated cells are enriched in genes related to the specification of the melanocytes, whereas the bipolar cells express NCSC markers. Interestingly, these NCSC-like cells display multilineage differentiation potential, including melanogenic differentiation, suggesting they may be an interfollicular reservoir for melanocytes (Fig. 2B).

Conversely, and complementary to the mouse, zebrafish melanocytes primarily reside in the skin (and lack hair follicles). Most embryonic skin melanocytes are directly derived from NCCs, but adult melanocytes within the stripe of the fish and fins originate from McSCs, and are dependent on ErbB-kinase signalling (the epidermal growth factor family of receptor tyrosine kinases) (Hultman et al., 2009; Parichy and Spiewak, 2015). Zebrafish McSCs replenish melanocytes during regeneration, as well as growth, and the manual or genetic ablation of McSCs causes large patches of skin that are devoid of melanocytes (Budi et al., 2008, 2011; Dooley et al., 2013; Hultman et al., 2009; Hultman and Johnson, 2010; Taylor et al., 2011). In embryos treated with an ErbB inhibitor, NCCs fail to migrate along the ventro-medial pathway and the peripheral nerves remain devoid of pigment progenitors (of which, the McSCs are a subpopulation) (Brombin et al., 2022; Budi et al., 2008, 2011; Dooley et al., 2013; Hultman et al., 2009). The impact of the ErbB inhibitor on melanocytes is maintained through to adulthood because zebrafish embryos treated with an ErbB inhibitor, or zebrafish with a hypomorphic mutation in *erbb3*, display large patches of skin without melanocytes (Budi et al., 2008; Dooley et al., 2013). Additional reservoirs of melanocyte precursors are associated with the endothelial compartment and the fin, suggesting that zebrafish have several McSC populations (Camargo-Sosa et al., 2019; Tu and Johnson, 2011).

Live imaging during zebrafish development has been able to reveal that McSCs reside in the DRG and give rise to melanoblast precursors. The melanoblasts migrate along nerves to the epidermis, where they differentiate into melanocytes (Budi et al., 2011; Dooley et al., 2013; Singh et al., 2016, 2014). In the adult, melanocytes and iridophores are primarily derived from nerve-associated progenitors, whereas xanthophores have a dual origin, and are also from an embryonic population that expands at the onset of morphogenesis (McMenamin et al., 2014; Singh et al., 2016). To characterise the molecular mechanisms that define the zebrafish DRG-associated McSCs, scRNA-seq, live imaging and lineage-tracing data over time led to the discovery that the transcription factor Tfap2b, and a select subset of its target genes, specify the ErbB-dependent multipotent McSC population. These *tfap2b*^+^ cells reside in the McSC niche and can generate all three pigment cell types and nerve-associated cells in adults (Brombin et al., 2022). Thus, *tfap2b* expression marks a multipotent adult stem cell in the zebrafish embryo (Fig. 2C). Once established, McSCs require the metabolic enzyme aldehyde dehydrogenase 2 (Aldh2) to transition from McSC quiescence to activation during regeneration. McSCs require purines for activation and to generate their progeny, and Aldh2 formaldehyde metabolism generates formate that is used by the one-carbon cycle as a purine source (Brunsdon et al., 2022).

In contrast to pathways that promote McSC activation in regeneration, *phosphatase of regenerating liver 3* (*prl3*) is required to restrain premature McSC activation. In a small molecule screen, a PRL3 inhibitor caused McSCs to become overactivated in regeneration (Johansson et al., 2020). Although apparently inactive in normal development, in regeneration, Prl3 functions with Ddx21 at transcription start sites to regulate transcription of endolysosomal vesicle pathway and maintain a stem cell-like state. Ddx21 is an RNA helicase that acts as a nucleotide sensor in the neural crest and melanoma (Santoriello et al., 2020; Sporrij and Zon, 2021). In processes conserved from zebrafish to humans, high levels of PRL3 prevent differentiation and are associated with stem-like states in melanoma (see below). Crucially, given the specific expression in regenerating cells and melanoma (and not in otherwise healthy tissues), PRL3 is a druggable target and a therapeutic antibody is in clinical trials for cancers with high levels of PRL3 (Chia et al., 2023; Thura et al., 2019).

As zebrafish transition from embryonic to adult stages, melanocyte cell numbers must be carefully coordinated with growing body size and skin expansion. One crucial hormone that coordinates morphogenic behaviours is thyroid hormone (TH), comprising thyroxine (T4) and its derivative triiodothyronine (T3), the ligand of nuclear thyroid hormone receptors. Fish that are 'hypothyroid' (due to transgenic ablation of the thyroid gland or mutations that reduce TH production) have about twice the normal number of melanocytes as wild type. By contrast, fish that are 'hyperthyroid' have fewer melanocytes, but more xanthophores, than wild type (McMenamin et al., 2014). scRNA-seq of NC-derived cells from embryo to adulthood, and other approaches revealed that TH promotes the maturation of both cell types. The hormone drives melanocytes to a terminally differentiated and proliferatively arrested state, which includes an acytokinetic mitosis that leads to multinucleation; in hypothyroid fish, melanocyte numbers are greater at least in part because relatively immature cells can continue to divide (Saunders et al., 2019). These findings in zebrafish may be relevant to human disease, because there is a high prevalence of hypothyroidism among individuals with uveal and cutaneous melanoma, suggesting that TH may function to limit melanocyte numbers in human tissues as well (Ellerhorst et al., 2003; Shah et al., 2006; Sisley et al., 1993).

In the adult zebrafish, melanocyte progenitors reside in the skin, supported by hypodermal cells (Aman et al., 2023), and replenish the adult stripe in regeneration by either differentiation or asymmetric cell division where one daughter differentiates while the other replenishes the progenitor cell (Iyengar et al., 2015). Using scRNA-seq combined with single-cell serial imaging in the adult skin, a recent study has identified two subpopulations of skin melanocyte progenitors present under normal conditions that contribute to pigmentation regeneration after ablation (Frantz et al., 2023). A third regeneration-specific population undergoes symmetric cell division and expresses markers associated with the multipotent pigment progenitors found in the embryo. This work indicates an unexpected melanocyte stem and progenitor cell heterogeneity in the adult skin, including regeneration-specific cell states.

## Melanocytes differ by anatomical site

The advent of single-cell sequencing technologies means the field is starting to gain insight into the heterogeneity of adult melanocytes at different anatomical sites in the skin. For example, two distinct versions of melanocytes have been identified from the human scalp and trunk tissues versus the foreskin, suggesting that different subpopulations may exist, or at least that epidermal melanocytes in different anatomical locations express genes that reflect differing microenvironments (Cheng et al., 2018). In a study of melanocytes from six human skin donors of European ancestry (ages 63-85), melanocytes have been shown to differ in mutation burden depending on anatomical site (Tang et al., 2020): melanocytes in the soles had few mutations, consistent with their sun-protected status; however, melanocytes in skin intermittently exposed to the sun, such as the back, thighs and shins, had more mutations than skin that is chronically exposed to the sun, such as the face. Although the donor numbers are small, this might indicate that there are differences in DNA damage repair between melanocytes depending on their anatomical locations. This may be relevant to the risk of melanoma because epidemiological data indicate that melanoma risk factors are associated with anatomical site. For example, intermittent UV light exposure sufficient to cause significant sunburn, increased density of nevi (non-cancerous moles) and very fair skin are risk factors for melanoma on the trunk, while chronic sun damage is associated with increased melanoma risk on the head and neck in pale skinned older people (Caini et al., 2009; Ghiasvand et al., 2019; Laskar et al., 2021; Mauguen et al., 2017; Siskind et al., 2005; Newton-Bishop et al., 2011).

Two additional key findings from the Tang et al. study suggest dynamic melanocyte processes yet to be understood. First, melanocytes from a single site exhibit a wide range of mutation burdens – surprising because melanocytes from the same site would have been expected to receive the same amount of UV exposure. One interpretation of these data is that melanocytes are replenished from sun-protected niches, such as the hair follicle, leading to heterogeneity in mutation burden among melanocytes within a single site. Second, shared mutations can be found among pairs and trios of melanocytes, suggesting that they stem from clonally related melanocytes (‘melanocyte fields') that may arise naturally over time through processes such as cell division, which has been observed in differentiated cells in zebrafish (Frantz et al., 2023; Iyengar et al., 2015; Taylor et al., 2011).

In a separate study, scRNA-seq on FACS-enriched (KIT^+^) melanocytes from 34 skin samples from around the body (22 donors; aged 9.5 fetal weeks to 81 years) showed that, despite differences in skin tone, sex and the individual donor, donor age was the primary clustering determinant across the samples (Belote et al., 2021). Interestingly, pigmentation and gene expression patterns in melanocytes from non-volar and volar (thick and hairless) skin in matched donor samples showed that melaninization in the non-volar human skin occurred between 12 and 18 weeks. The transcriptional differences between volar melanocytes and cutaneous non-volar melanocytes persist across donors of all ages, and these transcriptional signatures are found in acral melanoma (which arises on the palms and soles) and cutaneous melanomas, possibly reflecting distinct cells of origin for these melanomas. Positional information in the body is also crucial for determining the transformation potential of oncogenes in melanoma. Indeed, important work has shown that the anatomical location of melanocytes is crucial for oncogene specificity in acral melanoma (Weiss et al., 2022). Using DNA and RNA-seq of human patients, combined with functional and evolutionary genomics in zebrafish, has demonstrated that the limb/fin positioning gene *HOX13* synergises with the *CRKL* oncogene to generate a location-specific melanoma programme in acral melanoma. Thus, lineage-specific programmes cooperate with position-specific gene programmes to shape melanoma biology at different anatomical sites (Weiss et al., 2022).

## Dysregulation of the melanocyte lineage in melanoma

Melanoma is the deadliest form of skin cancer and originates from cells of the melanocyte lineage, including McSCs and differentiated melanocytes (Kohler et al., 2017; Moon et al., 2017; Sun et al., 2019). Despite improvements in targeted and immune-based therapies, many individuals still succumb to melanoma due to drug resistance, which can be the result of intratumour heterogeneity, generated by divergent genetic mutations, cellular transcriptional and metabolic diversity, and a complex tumour micro-environment (Kalaora et al., 2022; Karras et al., 2022; Lu et al., 2024; Rambow et al., 2019). Melanoma is well known for its high mutation burden, and mutations leading to increased MAPK signalling (including BRAF, RAS and NF1). However, dysregulated developmental lineages are generally now understood to be a significant driver of the evolution of tumour cell transcriptional heterogeneity (cell states) and the adaptive responses to therapy (Diener and Sommer, 2021). Molecular analysis of these dysregulated developmental states in cancer provides a rich source of new drug targets.

### Sox10 and the neural crest cell states in melanoma

Melanomas can be unpigmented, suggesting that the tumour cells comprise immature melanocytes. In zebrafish, for example, there is ample evidence of de-differentiation in melanoma towards a NCC-like state. In fact, the re-expression of NCC genes is crucial for the earliest stages of melanoma initiation: live imaging of individual cells over time labelled using the NCC-specific promoter *crestin* in zebrafish has shown that oncogene-expressing melanocytes are reprogrammed into NCC-like states before progressing into melanoma (Kaufman et al., 2016). Interestingly, *crestin* contains binding sites for transcription factors that are required for melanocyte specification (e.g. Sox10, Mitfa, Pax3 and Tfap2). Together, these studies suggest that reverting to a NCC-like state is required for melanocytes with oncogenic mutations to transition to melanoma. Furthermore, it has recently been shown that re-expression of Tfap2 proteins (a and e) in proliferative melanoma cells is necessary to cooperatively promote metastasis, indicating that NCC-like states are also necessary for cancer progression (Campbell et al., 2021).

In a similar way, as observed in NCCs in development, the NCC-like state in melanoma is sensitive to nucleotide depletion, and targeting this cell state with pyrimidine synthesis inhibitors leads to nucleotide depletion, and subsequent loss of NCC gene expression and inhibition of melanoma growth (Kaufman et al., 2016; Tan et al., 2016; White et al., 2011). Sox10 (which is a transcriptional regulator of NCCs) is also re-expressed in both human and mouse nevus melanocytes (nevi are commonly known as moles), which is indicative of de-differentiation to a NCC-like state. Notably, the loss of only one *Sox10* allele is sufficient to counteract the activity of oncogenes, such as *Nras* and *Grm1*, and to prevent melanoma in transgenic murine melanoma models (Cronin et al., 2013; Shakhova et al., 2012). Thus, melanoma depends on both the transcriptional and metabolic machinery of NCCs for initiation and growth.

Recently, a combination of zebrafish, human pluripotent stem cells and murine melanoma models have been used to demonstrate that cells within the melanocyte lineage have distinct oncogenic potential (‘permissively’) that is inversely correlated with their differentiation status. NCCs and melanoblasts express epigenetic factors that enable the expression of SOX10-regulated genes, creating an oncogenic potential cell state, compared with the relatively oncogene-resistant differentiated melanocytes (Baggiolini et al., 2021). Thus, a NCC developmental cell state is crucial for creating a permissive environment for oncogenic tumour initiation. Once melanomas are established, however, this lineage dependency on SOX10 may decrease because the loss of Sox10 activity is associated with drug resistance (Shaffer et al., 2017; Sun et al., 2014) and a cross-resistance mechanism for both targeted- and immune-based therapies (Purwin et al., 2023; Rosenbaum et al., 2023, 2021). This suggests that, as melanomas progress, they may become less dependent on established Sox10 NCC transcriptional networks and survive through alternative transcriptional cell states.

### MITF activity in melanoma

The levels of MITF in melanoma are complex, but generally as described by the ‘rheostat’ model: higher levels of MITF are associated with differentiation and proliferation, whereas lower levels are associated with invasion and drug resistance (Goding and Arnheiter, 2019). Both germline and somatic *MITF* mutations are associated with melanoma (Bertolotto et al., 2011; Dilshat et al., 2021; Garraway et al., 2005; Grill et al., 2013; Taylor et al., 2011; Yokoyama et al., 2011). Bulk RNA sequencing from melanoma cohorts has shown that both low and high levels of MITF activity are associated with poor outcomes (Cancer Genome Atlas, 2015; Lauss et al., 2016). Low MITF activity is especially crucial for individuals with melanoma because it is predictive of early resistance to targeted melanoma therapies (Muller et al., 2014). Indeed, de-differentiation of melanoma cells towards NCSC states (which have low MITF activity) is one mechanism in which melanoma cells escape T-cell detection and respond to inflammation, such as TNFα (Landsberg et al., 2012).

scRNA-seq experiments have shown that some tumour cell populations within melanoma tumours are more plastic (Marine et al., 2020). Single-cell profiling of both malignant and stromal cells derived from tumours of 19 individuals has confirmed that MITF is a key biomarker for distinct phenotypic states (Tirosh et al., 2016). These results reveal that MITF transcriptional activity is heterogenous, supporting a MITF^high^ (proliferative) and MITF^low^ (invasive) rheostat model (Bonet et al., 2017; Campbell et al., 2021; Goding and Arnheiter, 2019; Goodall et al., 2008; Hoek and Goding, 2010; Rambow et al., 2019). Indeed, *MITF* expression correlates with differentiation programmes and inversely correlates with the expression of the tyrosine kinase receptor-coding gene *AXL*; low MITF expression and high AXL expression are thus associated with the invasive, de-differentiated drug-resistance phenotypes. Notably, untreated MITF^High^ tumours contained some AXL^high^/MITF^low^ cells. The proportion of this population can be increased by treatment with a BRAF inhibitor (BRAFi) or BRAFi plus a MEK inhibitor (MEKi), designed to target the common BRAF mutations in melanoma that activate MAPK signalling, suggesting this population plays a crucial role in drug resistance. Together, these results highlight intra-tumour heterogeneity and show the challenges faced in melanoma to target both the MITF^high^ (proliferative) and MITF^low^ (invasive) phenotypic states that co-exist in the same tumour (Tirosh et al., 2016).

Single-cell analyses have been performed on human melanoma cells in a landmark study that captured cells through the course of therapy isolated from patient-derived xenograft mouse models following exposure to BRAFi (Rambow et al., 2018), revealing an even greater heterogeneity of MITF RNA and protein expression in melanoma. scRNA-seq profiling of the patient-derived xenograft tumours before, during and after therapy has identified at least four distinct melanoma cell states (clusters) associated with the minimal residual disease state (Rambow et al., 2018), melanoma cells that remain after drug treatment and give rise to disease recurrence (Travnickova et al., 2022). For example, a NC stem cell state has been characterised by the expression of a specific set (regulon) of genes (*SOX10*, *TFAP2B* and *MEF2C*) that are involved in NCC development (Rambow et al., 2018). Further scRNA-seq studies of mouse primary melanomas and human cancers have identified at least seven cellular states, with two states expressing genes associated with NCSCs (Karras et al., 2022). Although these two clusters share the expression of many genes, one cluster expresses more pluripotency factors and aligns more closely with a pre-migratory stem cell state described in mouse and avian neural crest, and may be stem cell-like cells that exist in a melanoma hierarchy to contribute to tumour growth (Karras et al., 2022; Kerosuo and Bronner, 2016; Soldatov et al., 2019). Pre-clinical mouse models of melanoma with immune checkpoint blockade has demonstrated that immunoresistance correlates with gene expression signatures found in embryonic undifferentiated melanoblasts, the hair follicle melanocyte stem cells and NC stem cell states, called melanoma plasticity signature (MPS) (Perez-Guijarro et al., 2020). Enrichment of this NC stem cell state (or MPS) in patients is predictive of immunotherapy resistance and associated with immune exclusion in melanomas (Boshuizen et al., 2020; Kim et al., 2021; Marine et al., 2020; Perez-Guijarro et al., 2020). Taken together with the studies described above, these studies suggest that melanoma cells de-differentiate towards the NCC lineage upon targeted- or immune-based therapies and, therefore, gain stem cell characteristics.

Zebrafish have been useful in dissecting some of the complex biology of the MITF ‘rheostat’. Using a conditional allele of MITF activity, it has been shown that low levels of MITF activity cooperates with BRAF and/or p53 mutations to promote melanoma, whereas turning off MITF completely leads to melanoma regression (Lister et al., 2014; Travnickova et al., 2019). scRNA-seq studies of cells from the primary tumour, residual disease site and the recurrent tumour have shown that MITF-dependent and MITF-independent cells co-exist in the primary tumour, and that MITF-independent cells survive in the residual disease site and contribute to recurrent disease (Travnickova et al., 2019). Interestingly, *mef2cb* and *tfap2b* are both genes that are expressed in McSCs in zebrafish, and their orthologues are expressed in residual disease cells (Rambow et al., 2018; Brombin et al., 2022), providing additional evidence of similarity between developmental and melanoma cell states.

Lineage dysregulation is not only limited to melanoma proliferation and therapy resistance but may also be required in melanoma metastasis. Although MITF^low^ cells are associated with stem- and invasive-like states, other melanocyte lineage programmes are dysregulated during melanoma metastasis. For example, the endolysosomal small GTPase Rab7 (normally involved in melanosome formation) is rewired early in melanomagenesis towards invasion (Alonso-Curbelo et al., 2014). In addition, RNA-seq analysis of migrating melanoblasts in development identified new gene expression signatures that re-emerge in late-stage metastatic melanoma called melanoma metastasis enhancer genes (MetDev genes), including the KDEL (Lys-Asp-Glu-Leu) endoplasmic reticulum (ER) protein receptor (KDELR) 3, which supports the ER-stress response and had not previously been known to be involved in metastasis (Marie et al., 2020).

## Conclusions

The advent of single-cell omic technologies has allowed us to interrogate the identity of thousands of cells in a single experiment, in unprecedented detail. As the molecular details of melanocytes across species are generated, these will help define melanocyte features unique to individuals, anatomic location, developmental stage, age and species, and, crucially, which of these can help identify new targets in disease, including melanoma, pigmentary disease and vitiligo. For example, although previous work has shown that McSCs in the hair follicle can repopulate regions of vitiligo, there remains an outstanding question of whether the interfollicular reservoirs of melanocyte progenitors can contribute to marginal re-pigmentation patterns, perhaps through cell division and differentiation, or through horizontal migration from pigmented areas of skin (Delmas and Larue, 2019; Lei and Hearing, 2020). Likewise, the impact of the melanocyte cell of origin and anatomical location on melanoma subtypes, or even mosaic pigmentary disease, is yet to be fully explored, but may provide new understanding of the aetiology of melanoma and unanticipated therapeutic targets (Belote et al., 2021; Kinsler and Larue, 2018; Kohler et al., 2017; Moon et al., 2017; Sun et al., 2019; Tang et al., 2020; Weiss et al., 2022). The recent advances in barcoding and lineage-tracing technologies could provide an experimental platform for deep characterisation of the origins of melanocytes in animal models and disease in an unbiased manner (Kalhor et al., 2018; Leeper et al., 2021; Raj et al., 2018a,b; Weng et al., 2024; Ye et al., 2020). In the future, it will be fascinating to follow how new lineage-tracing strategies (Travnickova et al., 2022) coupled with multi-modal technologies will extend our understanding of this enigmatic cell type, and explore the full contribution that NCCs, SCPs and McSCs – and possibly yet unknown sources – make to the melanocyte compartment throughout the human body.
